# Glucose-containing carriers in cancer therapy (GLUCOSE-ONC): A multistream critical review and quantitative exposure framework

**DOI:** 10.1016/j.neo.2026.101370

**Published:** 2026-09-22

**Authors:** Shinichiro Kashiwagi, Chika Watanabe, Yuki Asaka, Saeko Henmi, Hanae Matsuda, Huixian Ding, Yuko Kikukawa, Ayako Gose, Mai Nishimoto, Asuka Kochi, Koji Takada, Yukie Tauchi, Kana Ogisawa, Masatsune Shibutani, Hisakazu Fujita, Tamami Morisaki, Haruhito Kinoshita

**Affiliations:** a Department of Breast Surgical Oncology, Osaka Metropolitan University Graduate School of Medicine, 1-4-3 Asahi-machi, Abeno-ku, Osaka 545-8585, Japan; b Department of Gastrointestinal Surgery, Osaka Metropolitan University Graduate School of Medicine, 1-4-3 Asahi-machi, Abeno-ku, Osaka 545-8585, Japan; c Department of Scientific and Linguistic Fundamentals of Nursing, Osaka Metropolitan University Graduate School of Nursing, 1-5-17 Asahi-machi, Abeno-ku, Osaka 545-0051, Japan

**Keywords:** Chemotherapy, Dextrose, Glucose, Infusion, Metabolism, Tumour progression

## Abstract

Claims that glucose-containing anticancer infusions ‘feed cancer’ conflate a transient pharmaceutical carrier with chronic dysglycaemia and assume that glucose uptake equals tumour growth. We developed GLUCOSE-ONC, a multistream critical review separating direct systemic carrier comparisons, acute human glycaemia, parenteral nutrition as a high-exposure boundary, chronic dysglycaemia, and translational glucose–drug interactions. Four reproducible PubMed searches yielded 1,077 unique records; chronic dysglycaemia was handled as contextual evidence, and the clinically relevant set was curated with explicit causal-distance rules. Only one randomised systemic carrier comparison was identified: 5% glucose reduced gemcitabine vascular pain versus saline, but progression and survival were not measured. Dextrose-based hyperthermic intraperitoneal chemotherapy produced marked hyperglycaemia and inconsistent perioperative outcomes, but its large heated intraperitoneal exposure is not transportable to routine systemic carriers. Parenteral-nutrition trials, despite much larger and repeated nutrient exposure, showed mixed survival findings and no consistent neoplastic-acceleration pattern; infection and catheter effects contributed to net harm. Translational findings were bidirectional: high glucose induced resistance in some models but sensitised pancreatic and hepatocellular models, whereas glucose deprivation also induced resistance in selected endometrial and ovarian models. A 100–500 mL 5% dextrose bag contains 5–25 g; at 70 kg, 25 g over 2 h is 2.98 mg/kg/min, a transient flux subject to endogenous-production and disposal feedback. Standard glucose-containing infusion should not be conflated with chronic hyperglycaemia. Available evidence does not support the claim that transient carrier-level glucose exposure accelerates cancer progression, although direct outcome studies remain insufficient to prove exact equivalence.

## Highlights

• Carrier glucose exposure is finite and differs from chronic dysglycaemia.
• No direct study has linked routine dextrose carriers to cancer progression.
• Higher-dose parenteral nutrition shows no consistent acceleration pattern.
• Glucose–drug interactions are bidirectional across translational models.
• Definitive safety inference requires within-regimen causal comparisons.

## Introduction

The statement ‘glucose feeds cancer’ combines several true but non-equivalent observations into a clinical causal claim. Many cancers increase glucose uptake and reprogramme metabolism. Yet tracer uptake is not a direct measure of net tumour cell number, and metabolic competition also affects lymphocytes, myeloid cells and stromal compartments [1]. Demonstrating that a substrate enters malignant cells does not establish that a short additional exposure is rate-limiting, overcomes treatment-induced death, or changes progression over months.

Chronic hyperglycaemia is clinically important, but it is a host phenotype that can coexist with insulin resistance, inflammation, vascular disease, infection, glucocorticoid exposure, altered treatment intensity and adverse body composition. By contrast, 5% dextrose used as a pharmaceutical carrier is a finite exposure defined by grams, infusion time and compatibility. The two questions require different counterfactuals: sustained dysglycaemia versus euglycaemia, and the same anticancer regimen in dextrose versus a compatible glucose-free carrier.

Carrier choice is drug-specific rather than nutritional. Oxaliplatin requires 250–500 mL 5% dextrose and should not be mixed with chloride-containing solutions [2]. Fam-trastuzumab deruxtecan and datopotamab deruxtecan are diluted in 100 mL 5% dextrose and should not be diluted in sodium chloride [3,4]. Irinotecan and docetaxel permit dextrose or saline, whereas trastuzumab requires saline and prohibits 5% dextrose [[5], [6], [7]]. This pattern is consistent with stability and compatibility constraints, not a universal attempt to deliver glucose to tumours.

Direct progression studies were expected to be sparse. We therefore constructed GLUCOSE-ONC as a multistream review that preserves causal distance rather than pooling incomparable exposures. We asked whether routine systemic glucose-containing carriers have been shown to accelerate cancer; quantified their dose and flux; examined acute human glycaemia; used parenteral nutrition as a conservative, high-exposure stress test; separated hyperthermic intraperitoneal chemotherapy (HIPEC) and chronic dysglycaemia; and mapped bidirectional glucose–drug mechanisms. Figure 1 provides the symbolic exposure map; Table 1 summarises index carriers.

**Fig. 1:**
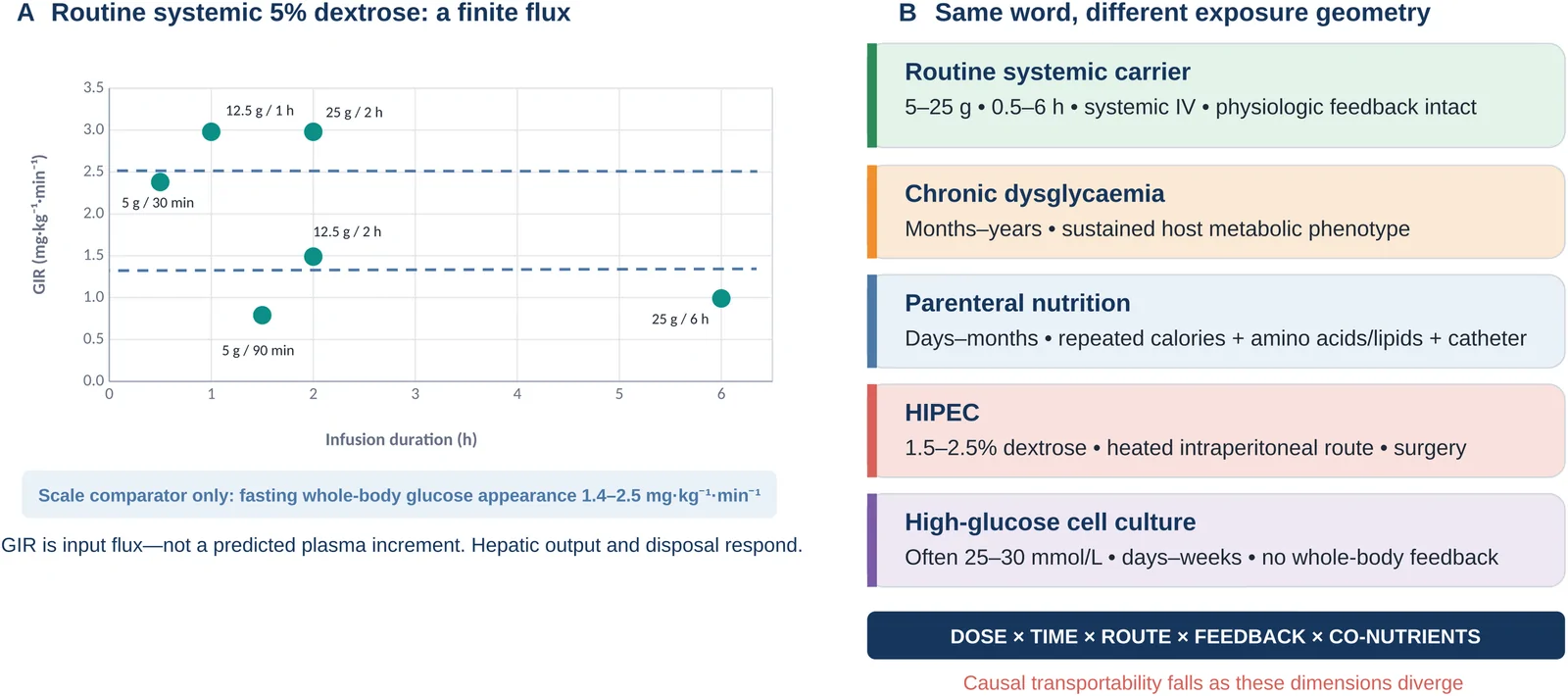
The bag is not the biology: quantitative exposure envelope and causal distance.

Panel A places labelled 5% dextrose carrier scenarios for a 70-kg patient against the reported 1.4–2.5 mg/kg/min fasting whole-body glucose-appearance range, used only as a scale comparator. Panel B separates routine systemic carrier exposure from chronic dysglycaemia, parenteral nutrition, HIPEC and high-glucose cell culture across duration, route, physiologic feedback and nutrient composition. GIR, glucose infusion rate; HIPEC, hyperthermic intraperitoneal chemotherapy; PN, parenteral nutrition.

**Table 1:** Index anticancer agents showing that carrier selection is drug-specific.

Agent	Label-compatible carrier	Volume, mL	Glucose, g	Typical infusion	Analytic role
Oxaliplatin	5% dextrose only; avoid chloride	250–500	12.5–25.0	2 h; may extend to 6 h	Mandatory compatibility anchor
Fam-trastuzumab deruxtecan	5% dextrose only; do not use saline	100	5.0	90 min first; 30 min if tolerated	Mandatory dextrose ADC
Datopotamab deruxtecan	5% dextrose only; do not use saline	100	5.0	90 min first; 30 min if tolerated	Mandatory dextrose ADC
Irinotecan	5% dextrose preferred or 0.9% saline	250–500	0 or 12.5–25.0	90 min	Within-agent comparison feasible
Docetaxel	0.9% saline or 5% dextrose	250	0 or 12.5	1 h	Within-agent comparison feasible
Trastuzumab	0.9% saline; do not use 5% dextrose	250	0	90 min loading; 30 min maintenance	Negative compatibility control

Abbreviations: ADC, antibody–drug conjugate. Labels vary by jurisdiction and date and must be verified before clinical use.

## Methods

### Review design and causal estimand

This is a structured multistream critical review with quantitative exposure modelling, not a completed protocol-registered systematic review. Reporting elements were informed by PRISMA 2020 and synthesis-without-meta-analysis guidance [[8], [9], [10]]. The target estimand is the effect on progression or death of initiating the same systemic anticancer drug or regimen in a standard glucose-containing carrier rather than a compatible glucose-free carrier, at a common treatment time zero. This explicit target-trial and causal-diagram formulation prevents chronic diabetes, caloric support and HIPEC from being treated as direct carrier evidence [11,12].

### Evidence streams, search and selection

Five evidence streams were defined a priori for causal classification: (1) human within-drug systemic carrier comparisons; (2) serial human glucose measurements around treatment; (3) controlled parenteral-nutrition studies as an upper-bound multi-nutrient exposure; (4) chronic dysglycaemia with oncologic outcomes; and (5) cell, organoid, animal or explant studies combining glucose contrast with anticancer treatment. HIPEC was retained only as a route-and-dose boundary. Progression or death was the primary causal outcome; glucose, response and toxicity were secondary.

On 27 August 2026, four PubMed strategies combined malignancy with carrier/dextrose terms (287 records), continuous glucose or acute hyperglycaemia terms (27), parenteral nutrition plus progression or survival (581), and high glucose or deprivation plus chemosensitivity or resistance (189). Deduplication by PMID yielded 1,077 unique records. Chronic dysglycaemia was treated as contextual evidence identified through targeted landmark retrieval and citation chaining rather than as a fifth systematic search. AI-assisted rules prioritised records for human review; no record was included or excluded solely by AI. Final eligibility decisions and data extraction were performed by authors. Product labels and citation chaining supplemented the map. Because no set of comparable direct progression estimates was found, no clinical effect estimate was pooled.

### Exposure-context and evidential hierarchy

Directness was ranked across dose, duration, route, host feedback, tumour, drug and endpoint. Tier 1 translational conditions approximated 4–11 mmol/L systemic glucose for ≤6 h or used a labelled carrier with measured patient glucose; Tier 2 represented >11–15 mmol/L or >6–24 h; Tier 3 represented ≥25 mmol/L culture, >48 h exposure, forced hyperglycaemic animals or deprivation. Parenteral nutrition and HIPEC remained boundary exposures and were never pooled with routine systemic infusion. Table 2 defines the streams and their causal role.

**Table 2:** Prespecified evidence streams and causal role.

Stream	Exposure contrast	Outcome	Role/directness
Direct systemic carrier	Same drug/regimen; dextrose vs compatible glucose-free fluid	Progression or survival	Highest directness; currently sparse
Acute human glycaemia	Serial glucose/CGM around treatment	Peak, time above range, iAUC	Moderate only if carrier and steroids recorded
Chronic dysglycaemia	Diabetes, HbA1c, fasting hyperglycaemia	Progression or survival	Context; duration and host phenotype differ
Parenteral nutrition	Repeated high-calorie multi-nutrient infusion	Response, recurrence, survival	Upper-bound stress test; net strategy effect
Translational	Glucose contrast plus anticancer drug in cells/organoids/animals	Sensitivity, resistance, mechanism	Plausibility; tier by concentration and time

Abbreviations: CGM, continuous glucose monitoring; iAUC, incremental area under the curve. HIPEC was retained as a route-and-dose boundary and was not pooled with routine systemic infusion.

### Quantitative exposure and systems model

For a 5% dextrose volume containing *D* grams delivered over *T* hours to a patient weighing *W* kilograms, the glucose infusion rate (GIR) was calculated as:

$$GIR\;=\;1000D\;/\;\left(60TW\right)\;mg\cdot \;kg^{-1}\cdot \;min^{-1}$$

D, glucose dose (g); T, infusion time (h); W, body mass (kg).

A minimal glucose-pool model was used to prevent the bag dose from being treated as a direct plasma increment:

$$dG/dt\;=R_{\text{exo}}\left(t\right)\;+\;EGP\left(G,I\right)\;-\;R^{d}\left(G,I\right)$$

EGP is suppressed and disposal rises as glucose and insulin change.

Instantaneous transport was stress-tested with *U(G)=V*max*G/(K*m*+G)*. This is deliberately an upper-bound transport calculation under fixed transporter capacity; it is not a tumour-growth model. Net volume was represented conceptually as *dV/dt=V[g(G,O₂, amino acids, perfusion, immunity)−k*drug*(C, redox)]*. Thus a progression claim requires evidence through multiple gates: systemic perturbation, tumour delivery, glucose limitation, durable biomass gain and dominance over drug-induced death (Figure 2).

**Fig. 2:**
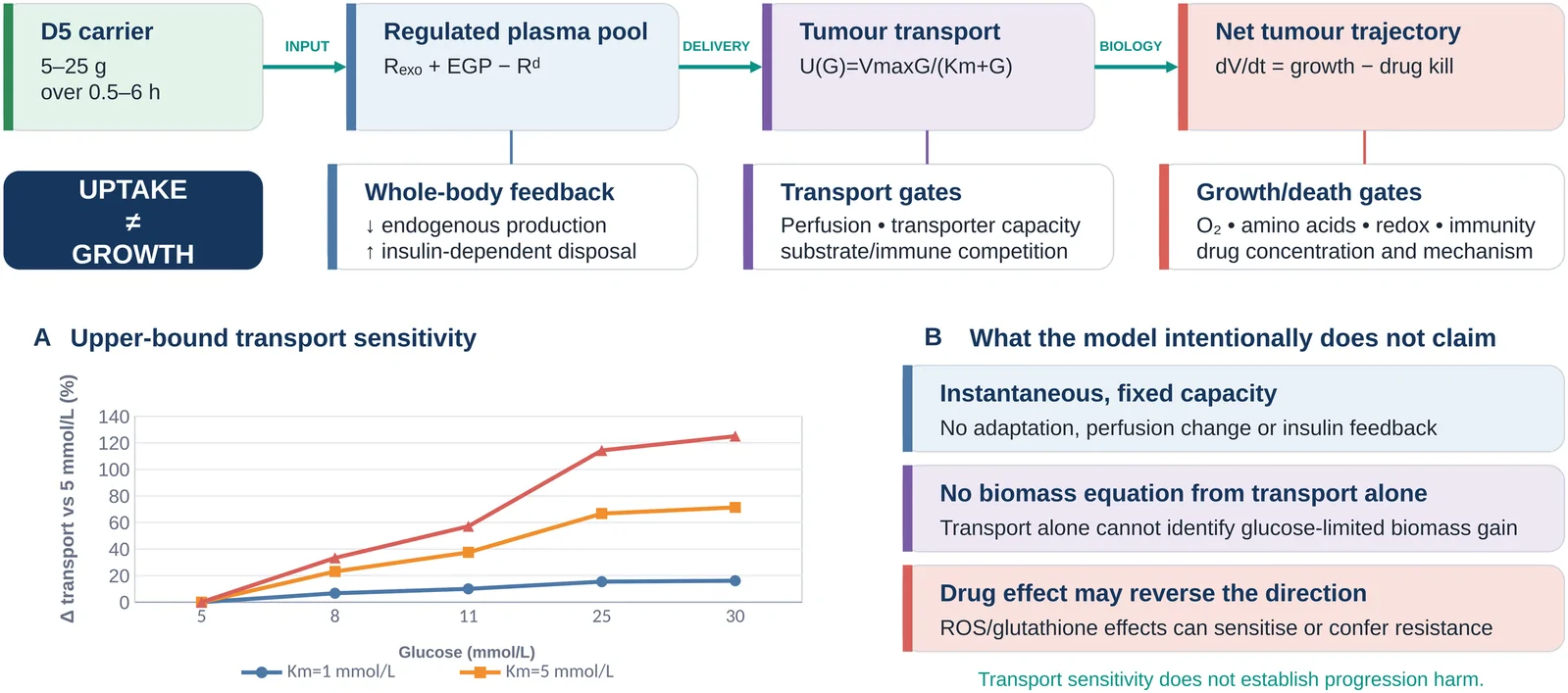
From infusion bag to tumour progression: a buffered causal system.

### Statistical decision framework

For design illustration, HR 1.10 was selected as a stringent candidate harm boundary, with HR 1.20 as a sensitivity analysis. These are analytic thresholds, not validated or regulator-endorsed non-inferiority margins; a definitive study would require clinical justification of its margin. If comparable direct studies emerge, the planned analysis is restricted maximum-likelihood random effects with Hartung–Knapp intervals and a prediction interval; a Bayesian model would report P(HR>1.10). Non-poolable evidence is grouped by stream and direction without vote counting. For an equal-allocation non-inferiority study under true HR=1, the approximate required number of progression-or-death events is:

$$D_{\text{events}}=4\;{\left(z_{1}-\alpha +z_{1}-\beta \right)}^{2}/{\left[log\left(\Delta \right)\right]}^{2}$$

One-sided α=0.025; Δ is the non-inferiority HR margin.

## Results

### Evidence map and direct systemic carrier evidence

The evidence map contained one randomised direct systemic carrier comparison and none with progression, response or survival as an outcome. In a Japanese double-blind crossover trial of 87 patients receiving weekly gemcitabine, 5% glucose versus saline reduced vascular-pain frequency (40% vs 63%) and mean intensity (1.3 vs 2.7; both p < 0.001) [13]. The study demonstrates that carrier randomisation is feasible and that clinically relevant non-oncologic effects can differ, but it cannot estimate cancer progression. Historical single-arm reports of prolonged 5-fluorouracil in 5% dextrose and intra-arterial BCNU likewise cannot isolate a carrier effect [14,15]. The correct result is therefore ‘not estimable’, not ‘harm’, and not proof of exact equivalence. Table 3 summarises the key human evidence across streams.

**Table 3:** Key human evidence. Boundary exposures are not pooled with routine systemic carriers.

Study	Design	Exposure	Key result	Carrier inference
Nagai 2013 [13]	Randomised crossover; n = 87	Gemcitabine in 5% glucose vs saline	Vascular pain 40% vs 63%; intensity 1.3 vs 2.7	No oncologic outcome
Torphy 2020 [16]	Retrospective HIPEC; n = 134	1.5% dextrose dialysate vs lactated Ringer’s	Severe hyperglycaemia 88% vs 21%; infection 35% vs 15%	Boundary exposure; no progression
Lindsey 2020 [17]	Retrospective HIPEC; n = 97	2.5% vs 1.5% dextrose perfusate	Peak glucose 199 vs 230 mg/dL; complications 20.8% vs 49.3%	Institutional-era confounding
McGeer 1990 [18]	Meta-analysis of controlled PN trials	PN during chemotherapy vs no PN	Survival RR 0.81; response OR 0.68; infection OR 4.1	Multi-component strategy; historical care
Nixon 1981 [19]	Randomised CRC; n = 50	Hypertonic glucose/amino-acid PN	Median survival 79 vs 305 days	Small trial; net PN effect
Nixon 1981 [20]	Randomised metastatic CRC; n = 45	PN adjunct to chemotherapy	No clinical or tumour-marker evidence of stimulation	Small trial; net PN effect
Vidal 2016 [21]	Randomised cystectomy cohort; n = 155 analysed	Postoperative PN vs no PN	Local recurrence 5.4% vs 4.9%; distant 31% vs 28%	PN; not a carrier comparison
Ulene 2025 [22]	Prospective CGM; n = 20	Chemotherapy plus dexamethasone	17% of readings ≥140 mg/dL; strong HbA1c gradient	Carrier not isolated

Abbreviations: CGM, continuous glucose monitoring; CRC, colorectal cancer; HIPEC, hyperthermic intraperitoneal chemotherapy; OR, odds ratio; PN, parenteral nutrition; RR, relative risk.

### HIPEC: a non-transportable route-and-dose boundary

HIPEC exposes a large peritoneal surface to heated perfusate during major surgery. In a retrospective 134-patient comparison, 1.5% dextrose dialysate versus lactated Ringer’s produced severe hyperglycaemia in 88% versus 21%, infections in 35% versus 15%, and an adjusted HR of 5.16 for infectious complications [16]. A separate institutional-era comparison of 2.5% versus 1.5% dextrose found similar peak glucose (199 vs 230 mg/dL; p = 0.15) and fewer overall complications with the higher concentration (20.8% vs 49.3%; p = 0.0143) [17]. In an uncontrolled cohort, 86% exceeded 180 mg/dL, glucose resolved by postoperative day 1 in 91%, and no association with postoperative outcomes was detected [23]. These findings support perioperative monitoring and demonstrate confounding across HIPEC cohorts. They do not measure progression and are not transportable to a 100–500 mL systemic carrier.

### Parenteral nutrition: a high-exposure stress test

A 1990 meta-analysis of controlled parenteral-nutrition trials reported trends toward lower survival (RR 0.81, 95% confidence limits 0.62–1.00) and response (OR 0.68, 0.40–1.10), with more infections (OR 4.1, 2.4–6.9) [18]. This is a net treatment-strategy effect involving repeated high calories, amino acids or lipids, hydration, central venous access, infection and patient selection; it does not isolate glucose-driven tumour growth.

Individual randomised trials in sarcoma, small-cell lung cancer, diffuse lymphoma, metastatic colorectal cancer, terminal cancer, gastrointestinal cancer and post-cystectomy populations were heterogeneous [[19], [20], [21], [24], [25], [26], [27]]. One 50-patient colorectal trial reported shorter median survival with total parenteral nutrition (79 vs 305 days), whereas another 45-patient trial found no clinical or tumour-marker evidence of neoplastic stimulation [19,20]. After cystectomy, local recurrence was 5.4% versus 4.9%, distant metastasis 31% versus 28%, and overall and cancer-specific survival did not differ [21]. A systematic review of 22 home-parenteral-nutrition studies involving 3,564 patients found substantial heterogeneity and limited causal comparators [28]. Much larger nutrient exposures therefore do not yield a consistent acceleration pattern, although procedure-related harms are real.

### Acute glycaemia and chronic dysglycaemia

In 20 women receiving early breast cancer chemotherapy with dexamethasone, all had at least one continuous-glucose-monitoring value ≥140 mg/dL, but only 17% of 124,165 readings were above that threshold. Time hyperglycaemic was 3.9%, 10.0% and 73.3% in baseline euglycaemia, prediabetes and diabetes, respectively [22]. The carrier was not isolated. This heterogeneity shows why HbA1c, diabetes, glucocorticoids, oral intake, stress and carrier must be measured separately.

A retrospective study of 157 patients with stage III colorectal cancer receiving adjuvant FOLFOX6 associated preoperative fasting glucose ≥126 mg/dL with worse disease-free and overall survival, accompanied by cell-line findings [29]. The exposure was baseline fasting glycaemia, not a measured carrier-induced excursion; a single baseline measurement also does not establish the duration of hyperglycaemia. This is relevant to metabolic care but cannot isolate the carrier effect because baseline dysglycaemia is a host characteristic subject to residual confounding by comorbidity, tumour biology, steroid exposure, treatment delivery and nutrition.

Pre-existing impaired glucose tolerance or diabetes may amplify or prolong the glycaemic response to a given glucose load. Accordingly, a short infusion need not produce an equally brief excursion in every patient. The HbA1c gradient above supports considering baseline glycaemic status as a potential effect modifier, although it does not quantify the carrier-specific response [22]. Chronic hyperglycaemia therefore remains relevant both to prognosis and to interpretation of acute exposure. Studies should measure baseline HbA1c and glucose-lowering treatment, then distinguish sustained glycaemic burden from the increment temporally associated with each infusion.

### Translational direction is bidirectional

Preclinical studies do not support a universal monotonic rule. Increased glucose availability sensitised pancreatic cancer to chemotherapy through lower GCLC/glutathione and greater oxidative damage [30], and sensitised hepatocellular carcinoma cells to doxorubicin and sorafenib through reactive-oxygen-species, DKK4 and P-glycoprotein effects [31]. Conversely, high glucose induced irinotecan resistance in colorectal models [32] and resistance in selected colon, glioblastoma, breast and haematologic systems. Glucose deprivation enhanced paclitaxel and doxorubicin effects in some breast cell lines [33], yet induced cisplatin resistance through SLC7A11 in endometrial cells and paclitaxel resistance after two weeks in ovarian cells [34,35]. Direction depends on drug mechanism, redox balance, glucose concentration and duration, oxygen, adaptation and lineage. Most experiments are Tier 3 and do not simulate a transient carrier. Prolonged high-glucose or deprivation conditions probe sustained metabolic stress and adaptation rather than the brief exposure of a routine infusion. These findings are retained solely as mechanistic context: neither resistance nor sensitisation in such models establishes the direction or magnitude of a clinical carrier effect. Translation requires experiments reproducing measured patient glucose trajectories, including return towards baseline between treatment cycles.

### Quantitative exposure context

Five-percent dextrose contains 5 g per 100 mL: 5 g in a 100-mL antibody–drug-conjugate bag, 12.5 g in 250 mL and 25 g in 500 mL. At 70 kg, 5 g over 90 min is 0.79 mg/kg/min, 12.5 g over 2 h is 1.49 mg/kg/min, 25 g over 2 h is 2.98 mg/kg/min and 25 g over 6 h is 0.99 mg/kg/min. A recent isotope study reported fasting whole-body glucose appearance of approximately 1.4–2.5 mg/kg/min in matched healthy volunteers [36]. This comparison supplies scale only: infused glucose is not simply added to endogenous production because hepatic output and peripheral disposal respond dynamically (Figure 1). The same GIR need not produce the same glucose trajectory in patients with different insulin sensitivity or secretory reserve. This scale comparison therefore neither predicts an individual glucose peak nor establishes metabolic safety in patients with impaired glucose tolerance.

Under fixed *V*max, the Michaelis–Menten stress test predicts that raising glucose from 5 to 8 mmol/L changes instantaneous transport by 6.7%, 23.1% or 33.3% for *K*m values of 1, 5 or 10 mmol/L; a rise from 5 to 11 mmol/L changes it by 10.0%, 37.5% or 57.1%. Even these deliberately permissive calculations do not predict biomass or progression: they fix transporter abundance and perfusion, omit substrate competition and immunity, and omit treatment-induced cell death.

### Net clinical context of mandatory-dextrose regimens

The established benefit of therapies that require dextrose is incompatible with the broad assertion that such treatment merely accelerates cancer. The 10-year MOSAIC analysis confirmed an overall-survival benefit for oxaliplatin-containing FOLFOX in stage III colon cancer [37], and large chemotherapy meta-analyses demonstrate disease-specific survival benefits [38]. These trials do not isolate a carrier effect, but they directly refute advice to abandon effective treatment solely because its compatible carrier contains glucose.

## Discussion

### Principal interpretation

The direct literature is too sparse to prove a zero carrier effect or definitively exclude HR 1.10. It is equally insufficient to support the affirmative claim of acceleration. Instead, the available evidence argues against conflation: the only randomised systemic carrier trial lacks oncologic outcomes; HIPEC is a different route and exposure; much larger parenteral-nutrition exposures show no consistent acceleration pattern; treatment-day glucose depends strongly on host and steroid context; and translational direction is bidirectional.

The principal conclusion is evidential rather than metaphysical: standard glucose-containing infusion should not be conflated with chronic hyperglycaemia. The available evidence does not support the claim that transient carrier-level glucose exposure accelerates cancer progression. ‘Does not support’ deliberately differs from ‘proves no possible effect’. This language is both clinically useful and proportionate to the evidence.

### Why glucose uptake is not tumour growth

At baseline, a tumour region may be limited by perfusion, oxygen, amino acids, redox capacity, immune pressure or drug damage rather than extracellular glucose. Exogenous glucose can suppress endogenous production and increase non-tumour disposal. Within the microenvironment, the same substrate is contested by cancer and immune cells, and chemotherapy interactions can reverse direction through oxidant or antioxidant pathways. A short change in substrate flux cannot be mapped to months-long progression without demonstrating tumour delivery, sustained rate limitation, durable biomass gain and dominance over drug-induced death. Figure 2 makes these gates explicit.

### Clinical implications

Carrier selection should follow the current product label, physicochemical stability, pharmacy policy, fluid and electrolyte needs, and patient-specific metabolic risk. Baseline diabetes, HbA1c, glucocorticoids and treatment-day glucose deserve attention because acute hyperglycaemia can affect infection and toxicity. These recommendations do not justify substituting an incompatible carrier. Patients should not stop chemotherapy, antibody–drug conjugates or other systemic treatment because a compatible bag contains glucose.

Glucocorticoids used for antiemetic or hypersensitivity prophylaxis are a separate contributor to treatment-associated hyperglycaemia. In a prospective study of 16 women without diabetes receiving carboplatin/paclitaxel with dexamethasone, 15 developed interstitial glucose >11.1 mmol/L; the duration varied substantially and was associated with baseline HbA1c [39]. This combined-treatment observation does not isolate the steroid or carrier contribution, but reinforces the need to record both exposures. Assessment should document steroid type, dose and timing, including oral doses before and after infusion, alongside carrier glucose and food intake. Monitoring should extend beyond the infusion when clinically indicated, because infusion duration does not define the entire period of metabolic exposure.

### A decisive multicentre study

A multicentre target-trial emulation is feasible in Japan because electronic prescribing and pharmacy-compounding systems record the actual carrier. Eligible patients would be new users of the same drug or regimen where both dextrose and saline are label-compatible, assigned at a common first-infusion time zero. Confounding control should include stage, tumour burden, HbA1c, diabetes, body mass index, cachexia, glucocorticoids, intake, organ function, performance status, dose intensity, site and calendar time. Overlap weighting with a doubly robust outcome model, restricted mean survival time, negative controls and site-by-calendar diagnostics would strengthen causal interpretation. Prespecified analyses should assess carrier effects by baseline glycaemic status and test interaction with HbA1c or diabetes. Steroid type, dose and timing should be standardised where feasible or explicitly accounted for, with serial glucose measurements used to characterise the resulting exposure rather than attributing all treatment-day hyperglycaemia to the carrier.

Small null studies will not be decisive. Under equal allocation, true HR=1 and one-sided α=0.025, excluding HR 1.10 requires approximately 3,457 progression-or-death events for 80% power and 4,627 for 90%; excluding HR 1.20 requires approximately 945 and 1,265 events, respectively (Figure 3). These are idealised event counts; exposure misclassification, residual confounding, clustering and limited covariate overlap would increase the required cohort size. The event requirement, not merely the patient count, should guide a national data network.

**Fig. 3:**
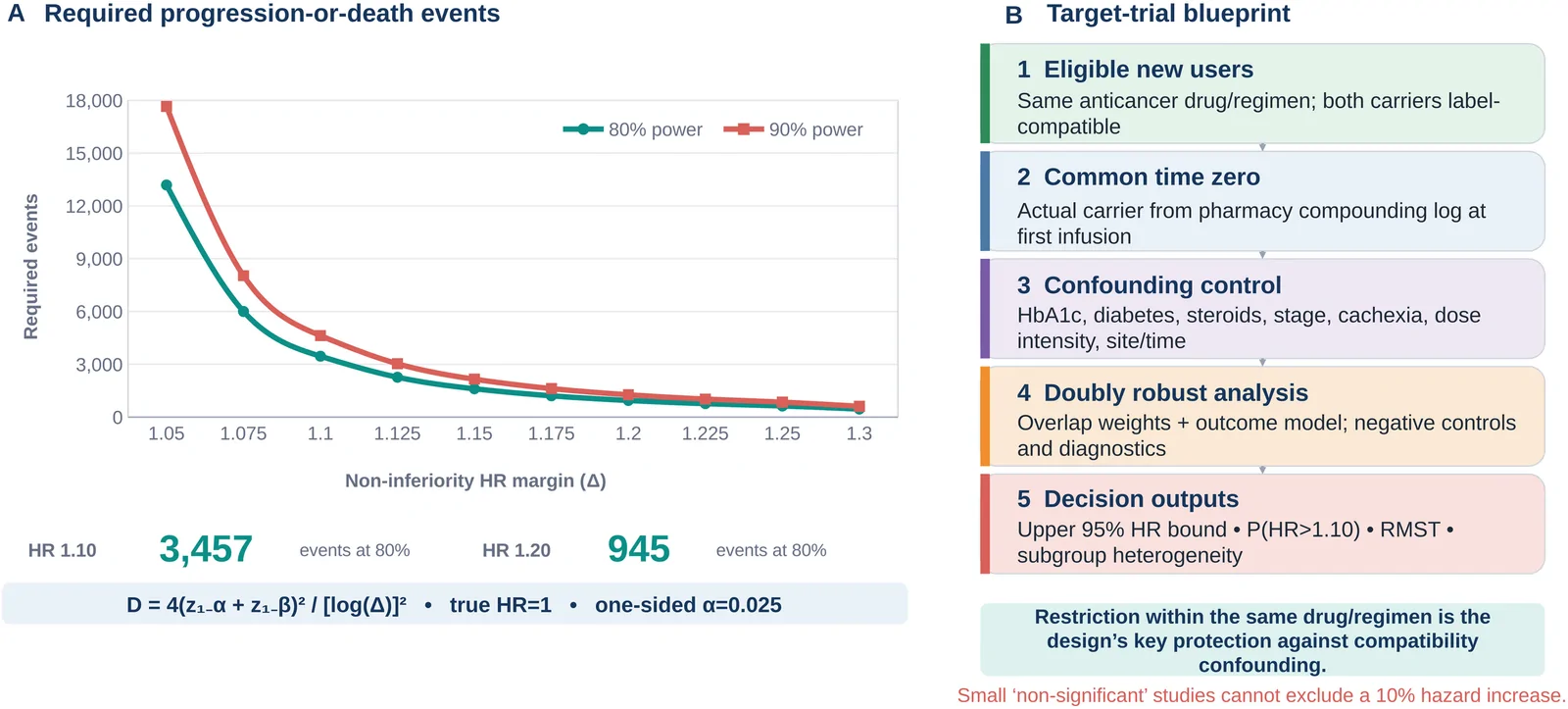
What evidence would exclude clinically relevant harm?.

Panel A shows the approximate progression-or-death event requirement for equal allocation, true HR=1 and one-sided α=0.025 across non-inferiority margins, with 80% and 90% power. Panel B gives a target-trial emulation blueprint for a multicentre study restricted to the same drug or regimen where both carriers are compatible. HR, hazard ratio; RMST, restricted mean survival time.

### Strengths and limitations

Strengths are the causal decomposition, preservation of evidence-stream boundaries, quantitative scale analysis, bidirectional mechanistic synthesis and explicit candidate harm thresholds. The main limitation is the near-absence of direct oncologic carrier comparisons. The search was PubMed-centred; chronic dysglycaemia was contextual rather than a fifth systematic search; and formal duplicate screening of every record, duplicate full-text extraction, comprehensive non-PubMed searching and consensus risk-of-bias assessment were outside the scope of this critical review. Accordingly, the work should not be interpreted as a completed systematic review. Older parenteral-nutrition reports often lack extractable hazard ratios and reflect historical supportive care. Label instructions vary by jurisdiction and date. The simple glucose-pool and transport equations are explanatory models, not patient-level pharmacodynamic validation. These limitations constrain certainty but do not create affirmative evidence for acceleration.

## Conclusions

Standard glucose-containing infusion should not be conflated with chronic hyperglycaemia. The available evidence does not support the claim that transient carrier-level glucose exposure accelerates cancer progression. This conclusion carries two safeguards: it is not proof of an exactly null effect, and it does not minimise chronic diabetes, steroid-associated hyperglycaemia, high-calorie parenteral nutrition or HIPEC-related metabolic complications. Compatible anticancer therapy should not be rejected on the basis of the unsupported ‘glucose carrier feeds cancer’ claim.

Future work should first establish the glucose trajectories produced by routine carriers in patients with normal and impaired glucose tolerance, using prospective serial sampling or continuous monitoring with complete steroid and nutritional records. Where both carriers are compatible, within-regimen randomised studies can separate the acute carrier contribution, followed by adequately powered oncologic comparisons as outlined above. Mechanistic studies should reproduce these measured, intermittent trajectories rather than sustained high-glucose culture or prolonged deprivation. A prospectively registered review with broader database coverage, duplicate selection and extraction, and formal risk-of-bias assessment would strengthen evidence completeness. These steps would address the present limitations in exposure attribution, experimental applicability and direct outcome evidence while preserving effective, compatible anticancer treatment.

## Ethics approval and consent to participate

Ethics approval and informed consent were not required because this review and quantitative exposure analysis used only aggregate data from published reports.

## Funding

This work was supported by the Japan Society for the Promotion of Science KAKENHI (JP20K08938 and JP23K08035). The funder had no role in study design, data collection, analysis, interpretation, writing, or the decision to submit.

## Declaration of generative AI and AI-assisted technologies in the manuscript preparation process

During the preparation of this work, the authors used ChatGPT (OpenAI) to support English-language editing, manuscript organisation, statistical-analysis planning, code drafting, and formatting review. After using this tool, the authors reviewed and edited the content as needed, independently reproduced and verified the analyses, code, text, and figures, and take full responsibility for the content of the article. No generative image model was used; the final scientific figures were constructed as deterministic, editable vector graphics from extracted study-level data and verified by the authors.

## Data availability

All study-level evidence used in the synthesis was derived from cited published aggregate reports; no individual-participant data were obtained and no new patient-level dataset was generated. Search dates, record counts, selection methods, equations and parameter values needed to reproduce the quantitative exposure calculations are reported in the manuscript and figures.

## CRediT authorship contribution statement

**Shinichiro Kashiwagi:** Writing – review & editing, Writing – original draft, Validation, Supervision, Methodology, Funding acquisition, Conceptualization. **Chika Watanabe:** Writing – review & editing, Validation, Investigation, Data curation. **Yuki Asaka:** Writing – review & editing, Validation, Investigation, Data curation. **Saeko Henmi:** Writing – review & editing, Validation, Investigation, Data curation. **Hanae Matsuda:** Writing – review & editing, Validation, Investigation, Data curation. **Huixian Ding:** Writing – review & editing, Validation, Investigation, Data curation. **Yuko Kikukawa:** Writing – review & editing, Validation, Investigation, Data curation. **Ayako Gose:** Writing – review & editing, Validation, Investigation, Data curation. **Mai Nishimoto:** Writing – review & editing, Validation, Investigation, Data curation. **Asuka Kochi:** Writing – review & editing, Validation, Investigation, Data curation. **Koji Takada:** Writing – review & editing, Validation, Investigation, Data curation. **Yukie Tauchi:** Writing – review & editing, Validation, Investigation, Data curation. **Kana Ogisawa:** Writing – review & editing, Validation, Investigation, Data curation. **Masatsune Shibutani:** Writing – review & editing, Validation, Methodology, Formal analysis. **Hisakazu Fujita:** Writing – review & editing, Validation. **Tamami Morisaki:** Writing – review & editing, Validation, Investigation, Data curation. **Haruhito Kinoshita:** Writing – review & editing, Validation, Investigation, Data curation.

## Declaration of competing interest

The authors declare that they have no known competing financial interests or personal relationships that could have appeared to influence the work reported in this paper.
